# B cell-mediated immune surveillance defines the favorable prognosis of occult breast cancer: a multi-omics study

**DOI:** 10.3389/fimmu.2026.1813674

**Published:** 2026-04-17

**Authors:** Junjie Liu, Rui Zhang, Ziyan Li, Xiaoqian Li, Xuetao Yuan, Jingyi Zhang, Na Chai, Yunpeng Zhang, Huimin Zhang

**Affiliations:** 1 Department of Breast Surgery, The First Affiliated Hospital of Xi’an Jiaotong University, Xi’an, Shaanxi, China; 2 Department of Dermatology, The First Affiliated Hospital of Xi’an Jiaotong University, Xi’an, Shaanxi, China; 3 Department of General Surgery, The First Affiliated Hospital of Xi’an Jiaotong University, Xi’an, Shaanxi, China; 4 Department of Pathology, The First Affiliated Hospital of Xi’an Jiaotong University, Xi’an, Shaanxi, China

**Keywords:** immune surveillance, multi-omics, occult breast cancer, prognosis, tumor microenvironment

## Abstract

**Background:**

Occult breast cancer (OBC) presents with axillary lymph node (ALN) metastases without detectable primary tumor (PT) yet exhibits paradoxically favorable prognosis compared to non-occult breast cancer (non-OBC). We aimed to elucidate mechanisms underlying PT clearance by integrating multi-omics approaches to characterize the unique immune landscape.

**Methods:**

Survival outcomes were validated using the Surveillance, Epidemiology, and End Results (SEER) database (n=12,162) with propensity score matching (PSM). Molecular features were characterized via quantitative proteomics, while immune landscapes were assessed using bulk transcriptomics. Single-cell RNA sequencing (scRNA-seq) and CellChat analysis dissected cellular heterogeneity and intercellular communication within tumor microenvironment.

**Results:**

Survival analysis confirmed OBC patients have survival comparable to T1N+M0 but significantly better than T2–3N+M0 breast cancer (BC) patients. Proteomic profiling identified B cell-related pathway upregulation in OBC lymph nodes (LN). Transcriptomics revealed enriched B cell infiltration in T1 tumors correlating with improved survival. scRNA-seq further demonstrated that B cells in T1 tumors act as central hubs in intercellular communication. These cells orchestrate a robust anti-tumor response via multi-faceted secretory signals (e.g., tumor necrosis factor, TNF; B-cell activating factor, BAFF) and contact-dependent interactions (e.g., CD40; major histocompatibility complex, MHC), effectively recruiting and activating immune effectors.

**Conclusion:**

Our findings suggest that B cell-mediated immune surveillance may be a key mechanism contributing to the clearance of PT in OBC. These exploratory results underscore the potential of B cells as prognostic biomarkers and therapeutic targets in BC, pending functional validation in larger cohorts.

## Introduction

OBC presents with ALN metastases in the absence of a detectable PT on imaging or pathological evaluation (1). Although the absence of a primary lesion complicates clinical staging, patients with OBC paradoxically exhibit a prognosis superior to that of patients with non-OBC (2, 3). Given that the conventional TNM staging system fails to explain this discrepancy, a thorough investigation into the pathogenesis of OBC is imperative to elucidate its unique biological behavior.

Detailed mechanistic exploration of OBC remains limited due to its low incidence (4). While a mammary origin is well-established—evidenced by the molecular profiles of metastases (5, 6) and favorable responses to local therapy (7–9)—the drivers of PT “occultation” remain obscure. Our previous proteomic study revealed that OBC exhibits enhanced epithelial-mesenchymal transition (EMT), accounting for its high metastatic propensity (3). However, the interplay between this aggressive phenotype and the immune system is poorly understood. Notably, OBC is unique among solid malignancies in presenting with LN metastases in the absence of a detectable PT. Consequently, OBC should be investigated not merely as a rare clinical entity, but as a unique natural model for elucidating mechanisms of human immune surveillance. A comprehensive investigation into these mechanisms holds significant scientific implications for the development of cancer immunotherapies.

In the present study, we aimed to elucidate the mechanisms underlying the “occultation” of the PT and the prognosis of OBC. We first leveraged a large-scale cohort from the SEER database to systematically validate the survival differences between OBC and LN-positive BC by T stage. Subsequently, we performed quantitative proteomic profiling to characterize the specific molecular features of OBC metastatic LN (OBC-LN). To further dissect the immune landscape, we integrated transcriptomic analyses from the METABRIC and in-house cohorts to evaluate immune cell infiltration, with a specific focus on B cell abundance. Finally, we employed scRNA-seq to unravel cellular heterogeneity and decode intercellular communication networks within the tumor microenvironment. By synthesizing these multi-omics approaches, we sought to demonstrate the critical role of B cell-mediated immune surveillance in the clearance of the PT, thereby providing a novel immunological perspective on the pathogenesis of OBC.

## Materials and methods

### SEER database

#### Data source and patient selection

The study was conducted in accordance with the Declaration of Helsinki and its subsequent amendments (10) and Strengthening the Reporting of Observational Studies in Epidemiology (STROBE) (11). We conducted a population-based retrospective cohort study utilizing the SEER database 17 (2000–2022), which was released in November 2024 and covers approximately 26.5% of the U.S. population. The inclusion criteria were as follows: female; age 20–89 years; diagnosis of a single primary BC (ICD-O-3 site code C50.9) between 2010 and 2022; definitive laterality (left or right); and known breast subtype (2010+). Tumor staging information was required based on the American Joint Committee on Cancer (AJCC) 7th edition (2010–2015) and the Extent of Disease (EOD) 8th edition (2018+) criteria for stage T0–3N1–3M0. To ensure data quality, we excluded patients with unknown race, those identified solely via autopsy or death certificate, and those with an overall survival (OS) of 0 months. A total of 12,162 eligible patients were identified, comprising 253 patients (T0N1–3M0) with OBC and 11,909 patients (T1–3N1–3M0) with non-OBC (**Supplementary Figure 1**).

#### Variants and endpoints

Data were extracted from the SEER database, including Patient ID, Survival months, Vital status recode, Age, Sex, Year of diagnosis, Site recode ICD-O-3/WHO 2008, Primary Site-labeled, T stage, N stage, M stage, ER Status, PR Status, HER2 status, Breast Subtype (2010+), Cause of death (COD) to site recode, Sequence number, Race recode (White, Black, Other), Year of follow-up recode, Type of Reporting Source, and Laterality. The primary endpoint was OS, defined as the time from diagnosis to death from any cause. BC subtypes were classified using the SEER registry variable “Breast Subtype (2010+),” which integrates ER, PR, and HER2 status for cases diagnosed since 2010. ER and PR status were derived from the variables “ER Status Recode Breast Cancer (1990+)” and “PR Status Recode Breast Cancer (1990+),” respectively, with hormone receptor (HR) positivity defined as ER- or PR-positive. HER2 status was determined using “Derived HER2 Recode (2010+).” Based on these markers, patients were categorized into four subtypes: HR+/HER2+, HR+/HER2-, HR-/HER2+, and HR-/HER2- (triple-negative breast cancer, TNBC). The coding of these variables follows pathology reports based on ASCO/CAP guidelines: ER/PR positivity is defined as immunohistochemical (IHC) staining in ≥1% of tumor nuclei; HER2 positivity is defined as IHC 3+ or gene amplification by *in situ* hybridization (ISH), while IHC 2+ or indeterminate ISH results are classified as equivocal.

### Statistical analysis

The Pearson chi-square test was used to compare demographic and clinicopathological characteristics between OBC and non-OBC groups. A 1:2 PSM was performed using the R MatchIt package (12). Nearest neighbor matching without replacement was employed, with the propensity score estimated via logistic regression. A caliper width of 0.3 standard deviations of the propensity score logit was applied, representing a compromise between minimizing bias (following Austin’s recommendation of 0.2 SD) (13) and maintaining an adequate sample size for the relatively small OBC group. Considering the impact of age on immune system competence and tumor-immune interactions, age was categorized into three subgroups (20–39 years, 40–59 years, and 60–89 years) during PSM to further eliminate the confounding effect of age. Covariates included age, year of diagnosis, N stage, ER status, PR status, HER2 status, breast subtype, race, and laterality. Standardized mean difference (SMD) was calculated to evaluate baseline balance. Survival was estimated using Kaplan-Meier survival analysis and hazard ratios (HRs) with 95% confidence intervals (CIs). Data processing and visualization were performed using R software (version 4.4.0) and RStudio (version 2023.9.1.494). P < 0.05 was considered statistically significant.

### Proteomic profiling

#### Tissue acquisition and processing

For proteomic analysis, six tissue samples were obtained from four treatment-naive BC patients at the Department of Pathology, Xi’an Jiaotong University. The samples comprised OBC-LN tissues from two patients, and primary tumors (non-OBC-PT) with paired metastatic LN (non-OBC-LN) from two non-OBC patients matched for baseline characteristics (**Supplementary Table 1**). Notably, both OBC patients presented with pathologically confirmed ALN metastases, yet no identifiable PT was found upon postoperative examination. All tissues were formalin-fixed and paraffin-embedded (FFPE), a preservation method validated to yield qualitative and quantitative proteomic profiles comparable to those of fresh-frozen samples (14). Proteins were extracted from FFPE tissues and analyzed by LC-MS/MS (**Supplementary Table 2**). The detailed experimental procedures for sample preparation, digestion, and mass spectrometry acquisition were identical to those reported in our previous study (3). Given the rarity of OBC, the proteomic profiling in this study was designed as an exploratory analysis.

#### Bioinformatics and statistical analyses

MS raw data were processed using DIA-NN software (version 1.8) for protein identification and quantification. A human protein database (UniProt, Homo_sapiens_9606_SP_20230103.fasta, containing 20,389 sequences) was used for database searching. The search parameters were set as follows: enzyme set to Trypsin/P with a maximum of 1 missed cleavage; fixed modifications included N-terminal methionine excision and carbamidomethylation of cysteine; the false discovery rate (FDR) for precursor and protein identification was set to 1%.

For data normalization, the normalized intensity values (I) obtained from the database search were subjected to centralization transformation to calculate the relative quantitative value (R) for each protein across samples, using the formula: R_ij_=I_ij_/Mean(I_j_), where i represents the sample and j represents the protein.

Differential expression analysis was performed using the R limma package to compare OBC-LN vs. non-OBC-PT and non-OBC-LN vs. non-OBC-PT. Differentially expressed proteins (DEPs) were defined using thresholds of |log_2_FC| > 0.5 and P < 0.05. Up- and down-regulated DEPs identified in OBC-LN and non-OBC-LN were intersected and visualized via Venn diagrams (R ggvenn package). Based on these results, DEPs were categorized into three subsets: OBC-LN-specific DEPs (up- or down-regulated), non-OBC-LN-specific DEPs (up- or down-regulated), and DEPs commonly up- and down-regulated in both OBC-LN and non-OBC-LN.

Gene Ontology (GO) and Kyoto Encyclopedia of Genes and Genomes (KEGG) pathway enrichment analyses were conducted on these subsets using the R clusterProfiler package. For GO enrichment, the analysis encompassed Biological Process (BP), Cellular Component (CC), and Molecular Function (MF) ontologies using the org.Hs.eg.db annotation database (version 3.19.1), which is based on the Gene Ontology snapshot dated January 17, 2024. The background gene universe for GO testing was defined as all Entrez Gene IDs with GO annotations in this package. For KEGG enrichment, the organism database was set to “human” referencing KEGG Release 116.0 (October 1, 2025), and the background gene universe consisted of all protein-coding genes annotated in the KEGG database for Homo sapiens (organism ‘hsa’). Multiple-testing correction was performed using the Benjamini-Hochberg (BH) method, with statistical significance defined as an adjusted P < 0.05.

Immune cell infiltration was quantified via single-sample gene set enrichment analysis (ssGSEA) using marker gene sets from Pornpimol et al. (15). Intergroup differences were assessed using the Kruskal-Wallis test (P < 0.05). Given the limited sample size (n=2 per group), these analyses were exploratory, and results were interpreted with caution due to limited statistical power.

### Transcriptome analysis

#### Data acquisition and cohort preparation

Transcriptomic data were obtained from two sources: the Molecular Taxonomy of Breast Cancer International Consortium (METABRIC; https://www.cbioportal.org/study/summary?id=brca_metabric), comprising 902 T1–3 stage (AJCC) BC patients with LN metastasis, available survival status, and documented tumor size; and an in-house cohort of 8 hormone receptor-positive, LN-positive patients diagnosed between 2018 and 2019 at the Department of Pathology, Xi’an Jiaotong University. The clinicopathological characteristics of the in-house cohort are detailed in **Supplementary Table 3**.

For METABRIC, gene expression was profiled using the Illumina HT−12 v3 microarray platform. In the original study, raw intensity data underwent background correction and quantile normalization, and were summarized to gene-level expression values, as detailed by Curtis et al. (16). The normalized expression matrix (log2 intensities) was obtained from cBioPortal. For the in-house cohort, RNA sequencing was performed at the Beijing Genomics Institute (Shenzhen, China) according to standard protocols. Gene expression levels were quantified as transcripts per million (TPM) and subsequently log2-transformed for downstream analyses.

#### Bioinformatics and statistical analyses

Immune cell infiltration was evaluated using ssGSEA and Tumor Immune Estimation Resource (TIMER) algorithms via the R GSVA and Immunedeconv packages (17). For ssGSEA, the marker gene sets were consistent with those described in the proteomics analysis section (Pornpimol et al. (15)). For TIMER, the analysis utilized immune cell-specific signatures defined by Li et al. (18), running with default parameters that incorporate tumor purity correction and specifying “BRCA” as the indication type. The abundances of six immune cell populations were estimated: B cells, CD4+ T cells, CD8+ T cells, neutrophils, macrophages, and dendritic cells. In the METABRIC cohort, statistical differences across T stages (T1a, T1b–c, and T2–3) were assessed using the Kruskal-Wallis test, with P-values adjusted for multiple testing using the BH method (adjusted P < 0.05). For the in-house cohort, given the limited sample size and the absence of T1a patients, differences between T1b–c and T2–3 stages were compared using the Wilcoxon rank-sum test (P < 0.05).

To investigate the prognostic impact of immune infiltration, optimal cutoff values for the METABRIC cohort were determined using the surv_cutpoint function in the R survminer package. Survival differences between groups were then compared using Kaplan-Meier analysis and HRs with 95% CIs, where P < 0.05 was considered statistically significant.

### Single-cell transcriptomic analysis

#### Data acquisition

scRNA-seq data was retrieved from the Gene Expression Omnibus (GEO: https://www.ncbi.nlm.nih.gov/geo/) database (GSE263995; accessed on January 6, 2026). In the current study, we focused on two treatment−naïve patients with clinically staged T1N+M0 (age 46 years; primary tumor size 16 mm; biopsy−proven ALN metastasis) and T2N+M0 (age 55 years; primary tumor size 25 mm; biopsy−proven ALN metastasis). Both patients received neoadjuvant chemotherapy with the cyclophosphamide (600 mg/m^2^) plus doxorubicin (90 mg/m^2^) regimen (on day 1 of each 21−day cycle) and subsequently achieved complete response according to standard criteria. For the T1N+M0 patient, samples from PT, matched metastatic LN, and peripheral blood (PB) were available (GSM8391107–GSM8391109); for the T2N+M0 patient, only a PT sample was profiled by scRNA−seq (GSM8208510). Given the limited number of available public specimens, the scRNA-seq analyses presented here were designed as exploratory and hypothesis-generating, aiming to identify candidate cellular mechanisms and interaction patterns that require validation in larger, independent cohorts.

#### Data processing and quality control

Raw scRNA-seq data were processed using the R Seurat package. Low-quality cells were excluded based on the following criteria: unique molecular identifiers (UMIs) < 100,000, detected gene counts outside the range of 300–7,000, mitochondrial gene percentages exceeding 10%, and hemoglobin gene percentages exceeding 3%. The filtered data were normalized using the “LogNormalize” method with a scale factor of 10,000. The top 3,000 highly variable features were identified via the “vst” method. Principal component analysis (PCA) was subsequently performed using the RunPCA function implemented in the R Seurat package. Based on the elbow plot evaluation, the first 20 principal components were retained for downstream analysis. Batch effects were corrected using the Harmony algorithm (19) with the sample origin (orig.ident) as the grouping variable. Subsequently, cells were clustered using a shared nearest neighbor (SNN) modularity optimization-based algorithm (FindNeighbors and FindClusters functions). The clustering resolution was set to 0.5, which was determined to be optimal for identifying major cell lineages in a dataset of this size (~35,000 cells), preventing over-clustering while ensuring clear separation of distinct populations. The results were visualized via Uniform Manifold Approximation and Projection (UMAP).

#### Cell type annotation

Cluster marker genes were identified using the Wilcoxon rank-sum test. Cell types were annotated based on canonical markers and established biological knowledge: T cells (CD3D, CD3E, CD3G), B cells (CD19, CD79B, MS4A1), fibroblasts (COL1A1, DCN, LUM), monocyte-derived dendritic cells (moDCs; S100A8, S100A9, CD68), natural killer (NK) cells (NKG7, KLRD1, KLRK1), proliferative cells (MKI67, CCNB1, CCNB2), macrophages (CD14, C1QB, C1QC), plasma cells (JCHAIN, IGHG1, XBP1), endothelial cells (PLVAP, VWF, CD34), epithelial cells (EPCAM, KRT8, KRT18), and platelets (ITGA2B, ITGB3, GP1BA).

#### Differential abundance analysis

To evaluate differences in B cell abundance across samples, a cell count matrix was constructed based on the annotated cell types. Differences in B cell abundance among the four samples—T1N+M0 (GSM8391107: PT; GSM8391108: LN; GSM8391109: PB) and T2N+M0 (GSM8208510: PT)—were evaluated using the Chi-square test implemented in the R stats package (function chisq.test). Subsequently, pairwise Chi-square tests were performed to identify specific sample pairs with significant differences. The resulting P-values from the pairwise comparisons were adjusted for multiple testing using the BH method (adjusted P < 0.05).

#### Cell-cell communication analysis

Cell-cell communication networks were inferred and compared using the R CellChat package (20). CellChat models signal communication between cells based on the law of mass action, quantifying the interaction strength as a communication probability by integrating gene expression data with prior knowledge of ligand-receptor interactions. The analysis utilized the CellChatDB.human database, specifically focusing on interactions within the “Secreted Signaling” and “Cell-Cell Contact” categories. For each sample (T1N+M0 and T2N+M0), overexpressed ligand-receptor interactions were first identified. Communication probabilities were then computed, and low-confidence interactions involving fewer than 10 cells were filtered out to ensure robustness.

To compare the communication landscapes between the T1N+M0 and T2N+M0 groups, CellChat objects were merged using the mergeCellChat function. Differential communication probabilities were calculated based on the aggregated network weights. The cell-cell communication heatmaps were generated using the netVisual_heatmap and netAnalysis_signalingRole_heatmap functions implemented in the R CellChat package, which utilize the R ComplexHeatmap package framework for visualization. In the comparative heatmaps, the color intensity represents the difference in communication probability or the relative contribution of signaling pathways between the two groups. This approach enabled the identification of differentially enriched ligand-receptor pairs and signaling pathways. The study specifically focused on extracting and comparing interactions where B cells acted as signal senders or receivers to elucidate their dynamic role in the tumor microenvironment.

## Results

### Survival differences between OBC and LN-positive BC by T stage

To compare the survival differences between OBC and LN-positive BC across different T stages, we identified 12,162 patients from the SEER database. The cohort comprised 253 OBC patients (median follow-up: 91 months) and 11,909 non-OBC patients (median follow-up: 52 months). The descriptive data for the study participants are presented in **Table 1** (before PSM) and **Table 2** (after PSM). As shown in **Table 1**, statistically significant imbalances were observed between the groups. Compared with the non-OBC group, patients with OBC were older (60–89 years: 50.6% vs. 37.3%) and diagnosed more frequently during the period 2010–2015 (76.3% vs. 55.4%) (both P < 0.001). Regarding pathological features, OBC was associated with more advanced nodal status (N3: 17.4% vs. 12.0%; P = 0.022) and distinct biomarker profiles. The OBC group exhibited significantly higher rates of ER-negative (32.4% vs. 17.0%) and PR-negative status (56.5% vs. 27.3%), alongside a lower prevalence of the HR+/HER2- subtype (50.2% vs. 70.1%) (all P < 0.001). No significant differences were observed regarding race or tumor laterality (P > 0.05).

**Table 1:** Clinicopathological characteristics of the patients from SEER dataset (before PSM).

Variants	Levels	non-OBC (N = 11,909)	OBC (N = 253)	P	SMD
Age	20–39 years	1387 (11.6%)	11 (4.3%)	<.001	.342
	40–59 years	6074 (51%)	114 (45.1%)		
	60–89 years	4448 (37.3%)	128 (50.6%)		
Year of diagnosis	2010-2015	6602 (55.4%)	193 (76.3%)	<.001	.451
	2016-2022	5307 (44.6%)	60 (23.7%)		
N stage	N1	8255 (69.3%)	170 (67.2%)	.022	.165
	N2	2229 (18.7%)	39 (15.4%)		
	N3	1425 (12%)	44 (17.4%)		
ER	Negative	2030 (17%)	82 (32.4%)	<.001	.362
	Positive	9879 (83%)	171 (67.6%)		
PR	Borderline/Unknown	20 (0.2%)	3 (1.2%)	<.001	.647
	Negative	3247 (27.3%)	143 (56.5%)		
	Positive	8642 (72.6%)	107 (42.3%)		
HER2	Negative	9486 (79.7%)	179 (70.8%)	<.001	.207
	Positive	2423 (20.3%)	74 (29.2%)		
Subtype	HR-/HER2-	1133 (9.5%)	52 (20.6%)	<.001	.437
	HR-/HER2+	779 (6.5%)	28 (11.1%)		
	HR+/HER2-	8353 (70.1%)	127 (50.2%)		
	HR+/HER2+	1644 (13.8%)	46 (18.2%)		
Race	Black	1504 (12.6%)	39 (15.4%)	.097	.144
	Other	1453 (12.2%)	21 (8.3%)		
	White	8952 (75.2%)	193 (76.3%)		
Laterality	Left	6054 (50.8%)	136 (53.8%)	.392	.058
	Right	5855 (49.2%)	117 (46.2%)		

**Table 2:** Clinicopathological characteristics of the patients from SEER dataset (after 1:2 PSM).

Variants	Levels	non-OBC (N = 505)	OBC (N = 253)	P	SMD
Age	20–39 years	22 (4.4%)	11 (4.3%)	.992	.01
	40–59 years	230 (45.5%)	114 (45.1%)		
	60–89 years	253 (50.1%)	128 (50.6%)		
Year of diagnosis	2010-2015	385 (76.2%)	193 (76.3%)	1.000	.001
	2016-2022	120 (23.8%)	60 (23.7%)		
N stage	N1	347 (68.7%)	170 (67.2%)	.908	.034
	N2	73 (14.5%)	39 (15.4%)		
	N3	85 (16.8%)	44 (17.4%)		
ER	Negative	166 (32.9%)	82 (32.4%)	.964	.01
	Positive	339 (67.1%)	171 (67.6%)		
PR	Borderline/Unknown	4 (0.8%)	3 (1.2%)	.865	.04
	Negative	288 (57%)	143 (56.5%)		
	Positive	213 (42.2%)	107 (42.3%)		
HER2	Negative	361 (71.5%)	179 (70.8%)	.900	.016
	Positive	144 (28.5%)	74 (29.2%)		
Subtype	HR-/HER2-	109 (21.6%)	52 (20.6%)	.986	.029
	HR-/HER2+	53 (10.5%)	28 (11.1%)		
	HR+/HER2-	252 (49.9%)	127 (50.2%)		
	HR+/HER2+	91 (18%)	46 (18.2%)		
Race	Black	78 (15.4%)	39 (15.4%)	.962	.021
	Other	39 (7.7%)	21 (8.3%)		
	White	388 (76.8%)	193 (76.3%)		
Laterality	Left	265 (52.5%)	136 (53.8%)	.798	.026
	Right	240 (47.5%)	117 (46.2%)		

To control for potential confounders, a 1:2 PSM was performed, resulting in a balanced cohort of 505 non-OBC patients and 253 OBC patients. Post-matching analyses (**Table 2**) confirmed that all baseline covariates—including age, year of diagnosis, N stage, hormone receptor status, molecular subtype, race, and laterality—were well-balanced between the two groups, with no statistically significant differences observed (all P > 0.05 and SMD < 0.05).

**Figure 1:**
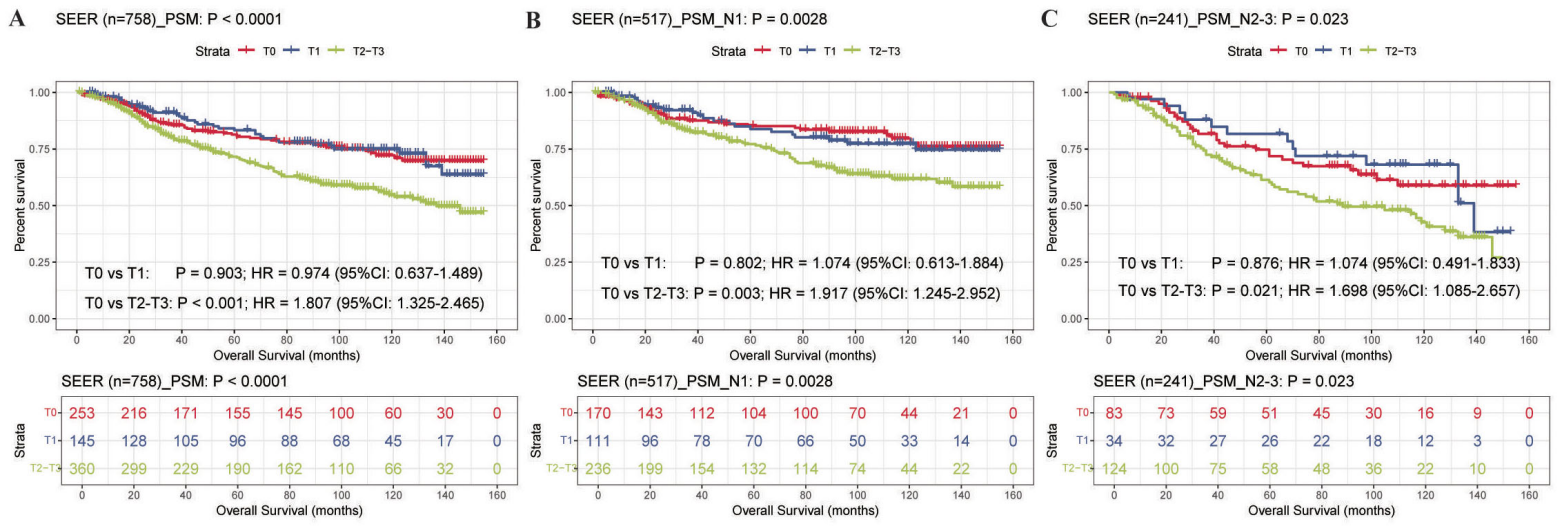
Kaplan-Meier survival analysis of OBC and LN-positive BC patients stratified by T stage after PSM. **(A)** Overall cohort (n=758; T0: n=253, T1: n=145, T2–3: n=360). **(B)** N1 subgroup (n=517; T0: n=170, T1: n=111, T2–3: n=236). **(C)** N2–3 subgroup (n=241; T0: n=83, T1: n=34, T2–3: n=124). PSM was performed using the R MatchIt package to balance baseline characteristics. PSM, propensity score matching; HR, hazard ratio; CI, confidence interval.

Survival analysis based on the PSM cohort revealed significant prognostic differences stratified by T stage (**Figure 1**). In the overall population (n = 758), patients with OBC (T0) demonstrated comparable survival outcomes to those with T1N+ BC (P = 0.903). However, OBC patients had significantly better survival compared to patients with T2–3N+ BC (HR = 1.807, 95% CI: 1.325–2.465, P < 0.001; **Figure 1A**). Subgroup analysis by LN status yielded consistent results. In the N1 subgroup (n = 517), no significant survival difference was found between T0 and T1 (P = 0.802), while T2–3 patients had significantly worse survival than T0 (HR = 1.917, 95% CI: 1.245–2.952, P = 0.003; **Figure 1B**). Similarly, in the N2–3 subgroup (n = 241), survival did not differ significantly between T0 and T1 (P = 0.876), whereas the T2–3 group exhibited a significantly higher risk of mortality compared to the T0 group (HR = 1.698, 95% CI: 1.085–2.657, P = 0.021; **Figure 1C**).

### Distinct proteomic profiles reveal specific B cell activation in OBC-LN

Our previous studies confirmed that OBC exhibits a higher propensity for ALN metastasis due to a pronounced EMT phenotype (3). To further elucidate the potential mechanisms underlying the “disappearance” of OBC-PT, we compared protein expression profiles between OBC-LN and non-OBC-PT, as well as between non-OBC-LN and non-OBC-PT.

**Figure 2:**
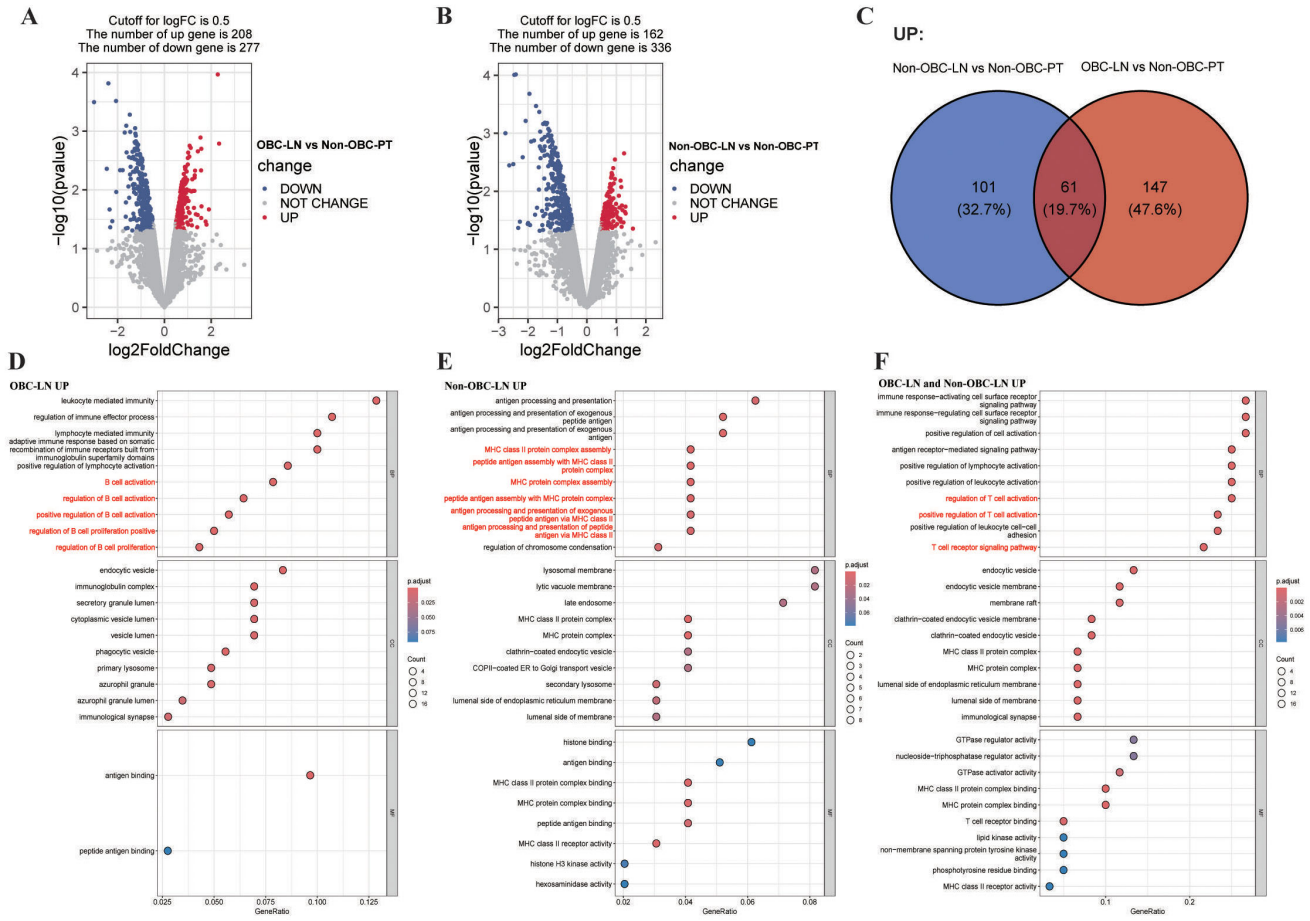
Comparative proteomic profiling and functional enrichment analysis of OBC-LN (n=2), non-OBC-LN (n=2) and non-OBC-PT (n=2). **(A, B)** Volcano plots showing the distribution of DEPs in OBC-LN vs. non-OBC-PT **(A)** and non-OBC-LN vs. non-OBC-PT **(B)**. DEPs were defined as |log_2_FC| > 0.5 and P < 0.05 using the R limma package. **(C)** Venn diagrams illustrating the unique and shared up-regulated DEPs between OBC-LN and non-OBC-LN. **(D-F)** Bubble plots displaying GO enrichment analysis of specific DEPs subsets. **(D)** OBC-LN-specific upregulated DEPs. **(E)** non-OBC-LN-specific upregulated DEPs. **(F)** Upregulated DEPs shared by both OBC-LN and non-OBC-LN. Statistical significance for pathway enrichment was defined as a BH adjusted P-value < 0.05.

Quantitative proteomics identified 485 DEPs in the OBC-LN vs. non-OBC-PT comparison (208 up-, 277 down-regulated) (**Figure 2A**), and 498 DEPs in the non-OBC-LN vs. non-OBC-PT comparison (162 up-, 336 down-regulated) (**Figure 2B**). Venn diagrams were subsequently constructed for the up- and down-regulated DEPs in both OBC-LN and non-OBC-LN groups (**Figure 2C**, **Supplementary Figure 2A**). Based on these intersections, the DEPs were categorized into three groups: 101 up- and 141 down-regulated DEPs specific to non-OBC-LN, presumed to mediate unique processes of non-OBC-LN metastasis; 147 up- and 84 down-regulated DEPs specific to OBC-LN, reflecting the molecular characteristics exclusive to OBC metastasis; and 61 up- and 193 down-regulated DEPs shared by both, representing common molecular events in BC-LN metastasis. GO enrichment analysis revealed that OBC-LN-specific up-regulated DEPs were primarily enriched in B cell-related pathways (**Figure 2D**, **Supplementary Table 4**), whereas non-OBC-LN-specific up-regulated DEPs were predominantly associated with MHC II-related pathways (**Figure 2E**, **Supplementary Table 5**). DEPs up-regulated in both groups were mainly enriched in T cell-related pathways (**Figure 2F**, **Supplementary Table 6**). Notably, no specific immune-related pathways were enriched among the corresponding down-regulated DEPs (**Supplementary Figures 2B–D**). These findings suggest that B cells may play a role in the clearance of OBC-PT. Furthermore, KEGG enrichment analysis revealed that OBC-LN-specific upregulated proteins were enriched in “Natural killer cell mediated cytotoxicity” and “Leukocyte transendothelial migration” pathways (**Supplementary Figure 3A**; **Supplementary Table 7**). ssGSEA further confirmed significantly elevated infiltration of myeloid-derived suppressor cells (MDSCs) in OBC-LN (P < 0.05; **Supplementary Figures 3B, C**). Collectively, these exploratory findings implicate B cells in the immune clearance of OBC-PT and shed light on potential immune mechanisms underlying the persistence of metastatic cancer cells in OBC-LN.

### B cell enrichment in T1-stage LN-positive BC correlates with improved prognosis

**Figure 3:**
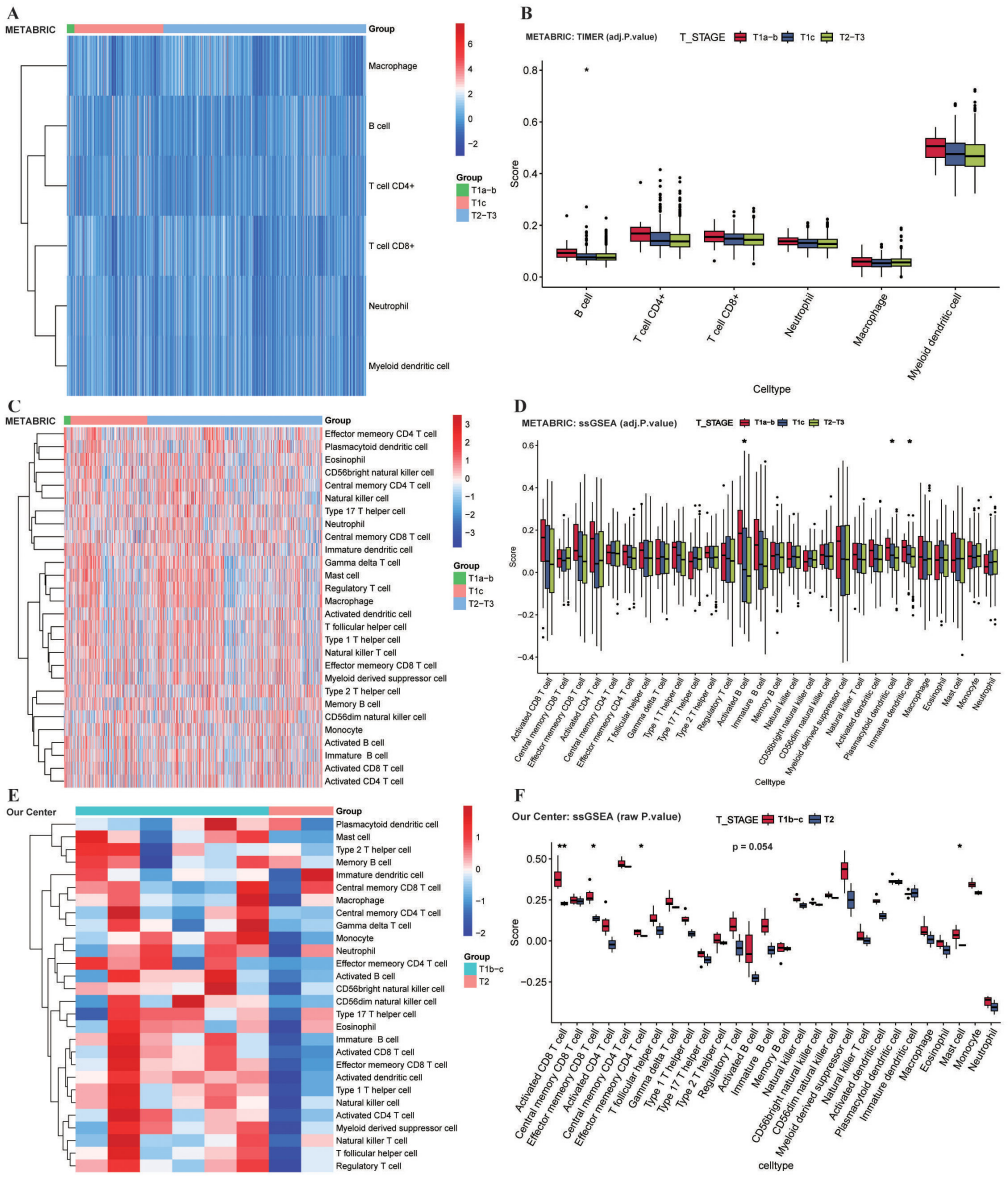
Comparative analysis of immune infiltration in T1 and T2–3 stages BC. **(A, B)** Infiltration levels of B cells in the METABRIC cohort (n=902) estimated by the TIMER algorithm. **(C, D)** Enrichment scores of activated B cells in the METABRIC cohort calculated by ssGSEA. **(E, F)** Validation of B cells infiltration trends in an in-house cohort (n=8) analyzed by ssGSEA. Statistical differences between T stage subgroups (T1a, T1b–c, T2–3) in **A–D** were assessed using the Kruskal-Wallis test, while differences between T1b–c and T2–3 in **E–F** were assessed using the Wilcoxon rank-sum test. P-values were adjusted using the BH method (adjusted P < 0.05). TIMER, Tumor Immune Estimation Resource; ssGSE, single-sample gene set enrichment analysis; * indicates: P < 0.05; ** indicates: P < 0.01.

**Figure 4:**
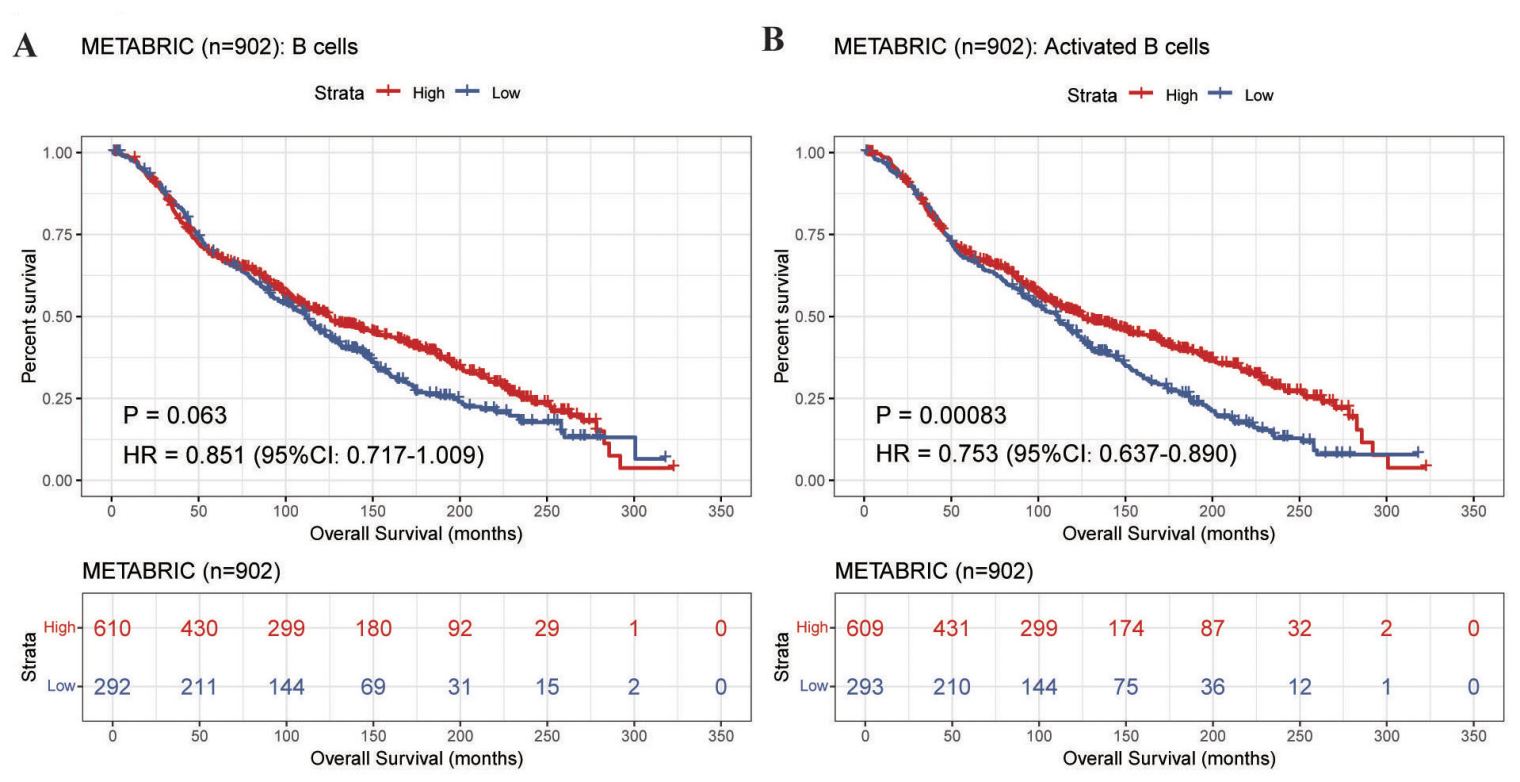
Prognostic value of B cells infiltration in the METABRIC cohort (n=902). **(A)** Kaplan-Meier survival curves stratified by total B cells infiltration levels. **(B)** Kaplan-Meier survival curves stratified by activated B cells infiltration levels. Patients were dichotomized into high- and low-infiltration groups using optimal cut-off values determined by the R survminer package.

To decipher the biological mechanisms underlying PT “occultation” in OBC, we sought a clinically relevant surrogate model given the inherent inaccessibility of the OBC-PT. Based on the distinct prognostic equivalence between OBC and T1N+M0 BC established by our SEER analysis, we hypothesized that T1N+M0 BC represents an immunological phenotype analogous to OBC, characterized by an active surveillance milieu capable of suppressing tumor outgrowth. To validate this hypothesis and uncover the drivers of PT clearance, we analyzed transcriptomic data from 902 LN-positive BC patients (stages T1–3) in the METABRIC cohort to systematically compare immune landscapes between small (T1) and large (T2–3) PT burdens. TIMER analysis revealed significantly higher B cell infiltration in T1a-b patients compared to other stages (adjusted P < 0.05; **Figures 3A, B**). This was further confirmed by ssGSEA, which demonstrated a greater abundance of activated B cells in the T1a-b subgroup (adjusted P < 0.05; **Figures 3C, D**). Validation in an in-house cohort showed a similar trend of elevated activated B cells infiltration in T1 patients (P = 0.054; **Figures 3E, F**). Note that for the in-house cohort, given the limited sample size (n=8) and the focus on a single pre-specified comparison (T1b-c vs. T2-3), P-values are presented without adjustment for multiple testing, and the result should be interpreted with caution. These findings corroborate our OBC results, suggesting that B cells may participate in the clearance of PT in both OBC and T1N+ BC. Furthermore, Kaplan-Meier survival analysis indicated that high levels of activated B cell infiltration were associated with significantly improved OS (HR = 0.753, 95% CI: 0.637–0.890, P < 0.001; **Figures 4A, B**).

### Single-cell dissection of immune cell composition and interactions in T1 vs. T2 LN-positive BC

**Figure 5:**
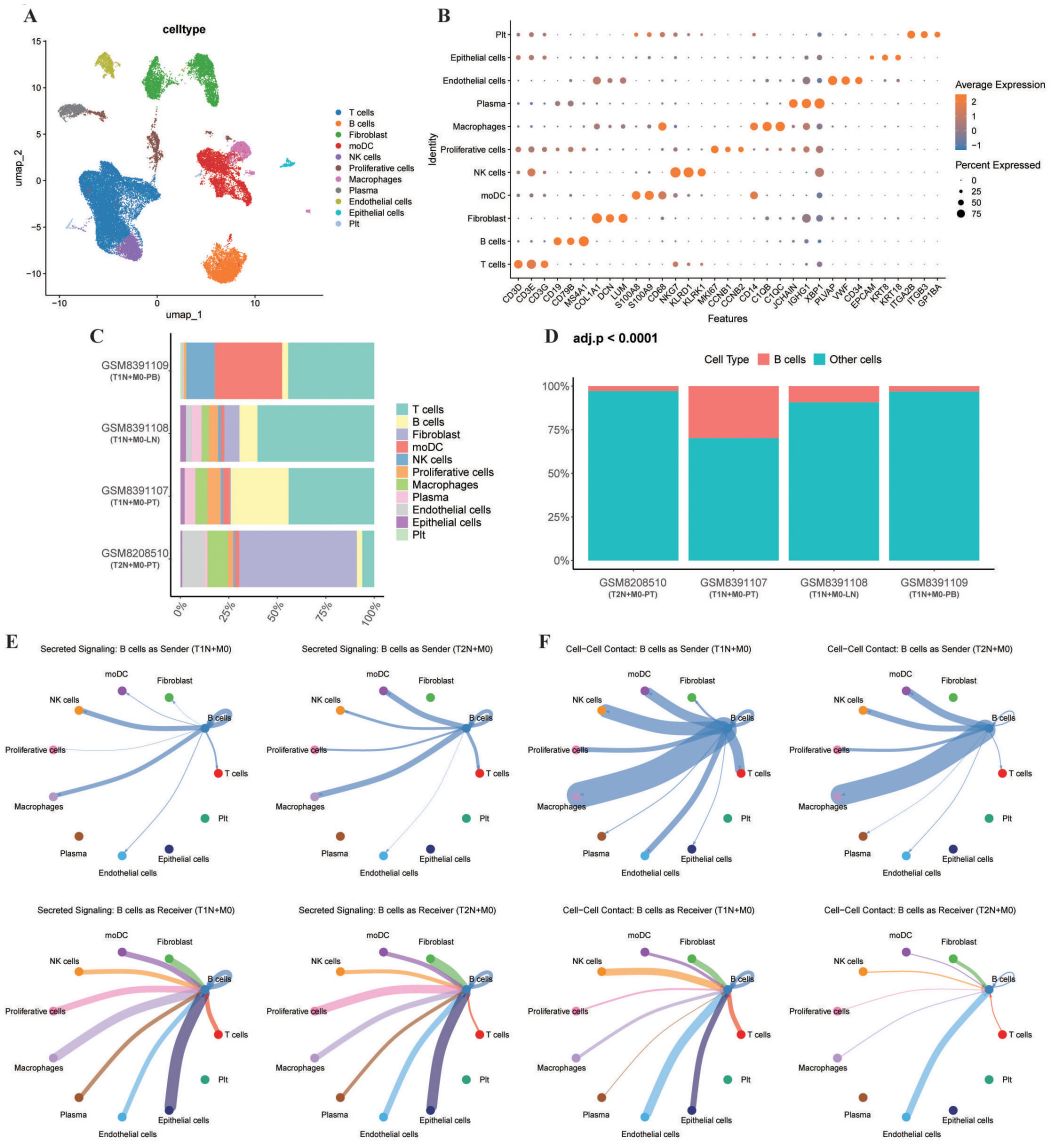
Single-cell dissection of immune cell composition and interactions in T1N+M0 vs. T2N+M0 BC. **(A)** UMAP visualization of 34,530 high-quality cells from two patients (four samples), clustered into 20 distinct cell populations. **(B)** Dot plot showing the average expression and percentage of cells expressing canonical marker genes for each cell type. **(C)** Stacked bar plot depicting the cellular composition of each sample. **(D)** Comparison of B cell proportions between T1N+M0 and T2N+M0 PT; statistical significance was determined by the Chi-square test with BH adjustment (adjusted P < 0.0001). **(E)** CellChat analysis of secreted signaling pathways, with B cells as senders (top) and receivers (bottom) in T1N+M0 (left) and T2N+M0 (right) PT. **(F)** CellChat analysis of cell-cell contact pathways, with B cells as senders (top) and receivers (bottom) in T1N+M0 (left) and T2N+M0 (right) PT. PB, peripheral blood; LN, lymph node; PT, primary tumor.

To dissect the cellular heterogeneity and interaction networks underlying the tumor immune microenvironment of T1 and T2 LN-positive BC, we performed scRNA-seq analysis on samples from two treatment-naive TNBC patients: a T1N+M0 patient (PT, metastatic LN, and PB) and a T2N+M0 patient (PT). After quality control (**Supplementary Figures 4A, B**), a total of 34,530 high-quality cells were retained and clustered into 20 distinct cell populations (**Supplementary Figure 4C**). Cell type annotation based on canonical marker genes identified T cells, B cells, fibroblasts, moDCs, NK cells, proliferative cells, macrophages, plasma cells, endothelial cells, epithelial cells, and platelets (**Figures 5A, B**). Notably, the proportion of B cells was significantly higher in the T1N+M0 PT compared to the T2N+M0 PT (**Figures 5C, D**; adjusted P < 0.05), consistent with our bulk transcriptomic findings. These observations should be interpreted as exploratory, given that only two patients (four samples) were included. Using the CellChat algorithm, we analyzed cell-cell communication networks, focusing on secreted signaling and cell-cell contact pathways. In T1N+M0 PT, B cells exhibited enhanced communication as both signal senders and receivers (**Figures 5E, F**). Specifically, B cells in T1N+M0 showed increased interaction with T cells, NK cells, Macrophages, Epithelial cells and moDCs via secreted signaling pathways, while cell-cell contact pathways were also more prominent. These results suggest that B cells in T1N+M0 PT play a more active role in modulating the tumor microenvironment through direct and indirect interactions with other immune and stromal cells.

### Differential ligand-receptor interactions reveal B cell-mediated anti-tumor mechanisms in T1 vs. T2 LN-positive BC

**Figure 6:**
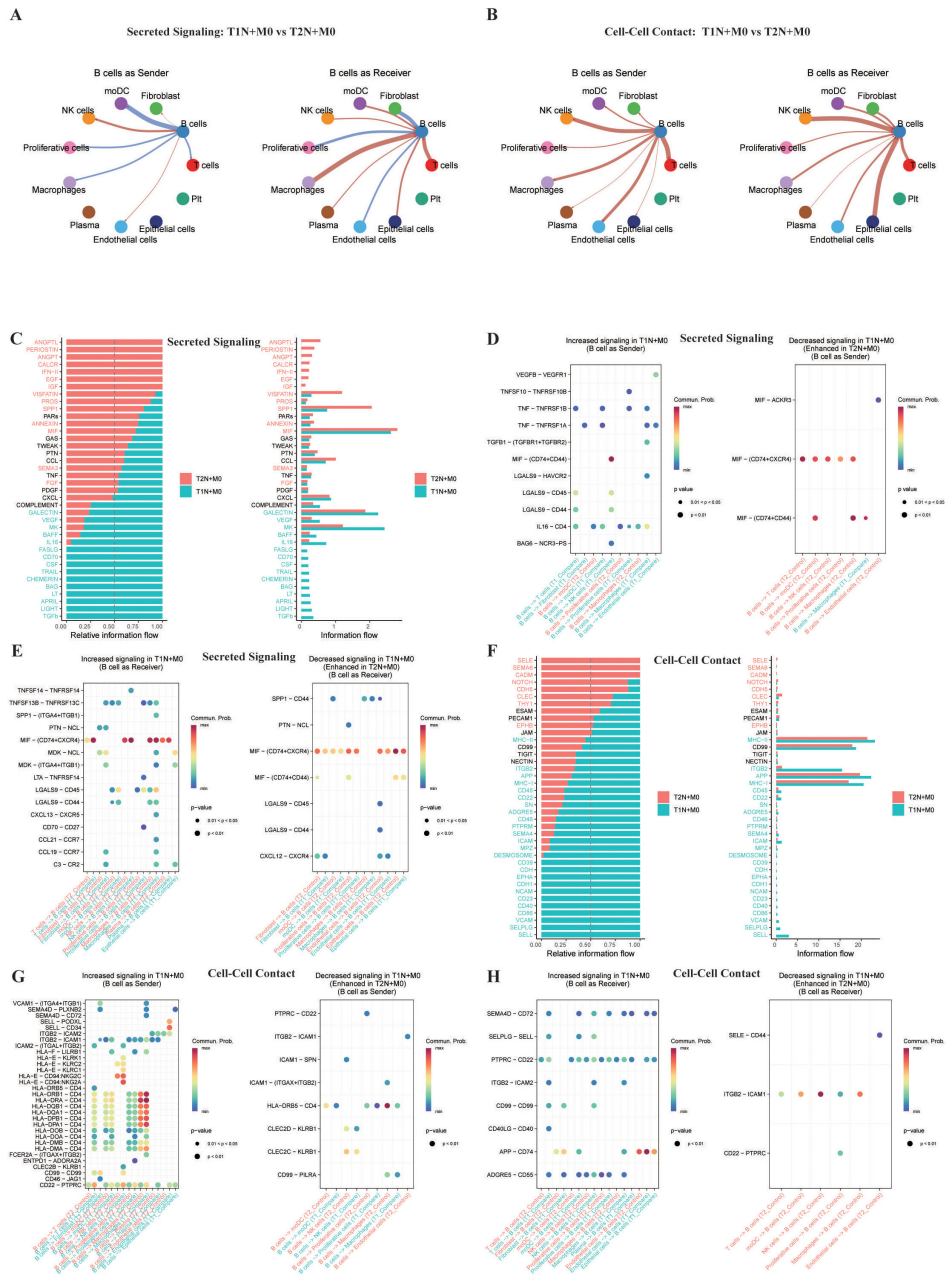
Differential ligand-receptor interactions in T1N+M0 vs. T2N+M0 BC. **(A)** Differential network analysis of secreted signaling, depicting B cells as senders (left) and receivers (right). **(B)** Differential network analysis of cell-cell contact, depicting B cells as senders (left) and receivers (right). **(C)** Comparative ranking of secreted signaling pathways between T1N+M0 and T2N+M0. **(D)** Bubble plot of ligand-receptor pairs with B cells as senders in secreted signaling, showing increased (left) and decreased (right) interactions in T1N+M0. **(E)** Bubble plot of ligand-receptor pairs with B cells as receivers in secreted signaling, showing increased (left) and decreased (right) interactions in T1N+M0. **(F)** Comparative ranking of cell-cell contact pathways between T1N+M0 and T2N+M0. **(G)** Bubble plot of ligand-receptor pairs with B cells as senders in cell-cell contact, showing increased (left) and decreased (right) interactions in T1N+M0. **(H)** Bubble plot of ligand-receptor pairs with B cells as receivers in cell-cell contact, showing increased (left) and decreased (right) interactions in T1N+M0.

To dissect the heterogeneity of intercellular communication within the tumor microenvironment between T1 and T2 LN-positive BC, we employed CellChat to perform a comprehensive differential analysis of ligand-receptor interactions. This reorganization manifested as divergent communication patterns: in secreted signaling, B cells in T1N+M0 PT showed enhanced interactions with NK cells as senders, whereas B cells in T2N+M0 favored moDCs; as receivers, T1N+M0 B cells engaged more with T cells and macrophages, while T2N+M0 B cells interacted more with stromal cells (endothelial cells, fibroblast) (**Figure 6A**, **Supplementary Figure 5A**). In cell-cell contact, B cells in T1N+M0 exhibited significantly enhanced direct communication with immune effectors (T cells, NK cells) regardless of their sender/receiver role (**Figure 6B**, **Supplementary Figure 5B**).

Focusing on secreted signaling, a comparative ranking of global information flow revealed that the T1N+M0 microenvironment is significantly enriched for immune-activating networks compared to T2N+M0 (**Figure 6C**). Specifically, T1N+M0 exhibited a marked upregulation of B cell-specific regulatory circuits, including BAFF and a proliferation-inducing ligand (APRIL), pivotal for B cell survival and maturation. Other TNF superfamily members were also upregulated, such as TNF, a potent pro-inflammatory cytokine, and TNFSF14 (LIGHT), a co-stimulatory molecule that enhances immune cell activation, whereas T2N+M0 showed a relative predominance of stromal signaling (e.g., CXCL12-CXCR4, which mediates immunosuppressive cell recruitment). At the ligand-receptor pair level, B cells in T1N+M0 acted as potent senders of pro-inflammatory signals, utilizing TNF, macrophage migration inhibitory factor (MIF)-CD74-CD44, and galectin-9 (LGALS9), a molecule involved in immune cell adhesion and activation, to engage immune effectors (**Figure 6D**). Conversely, as receivers, B cells in T1N+M0 were immersed in a niche conducive to organization and activation, receiving strong chemotactic signals via CXCL13-CXCR5, (CCL19/21)-CCR7, as well as co-stimulatory inputs via BAFF and CD70, a ligand for the costimulatory receptor CD27, along with complement pathways (**Figure 6E**). This suggests that the T1N+M0 niche actively supports B cell recruitment, maturation, and effector differentiation.

Beyond secreted signaling pathway, we extended our analysis to cell-cell contact. The T1N+M0 cohort displayed a robust enrichment of pathways critical for antigen presentation and immune synapse formation, including major histocompatibility complex class I (MHC-I), MHC-II, essential for antigen presentation, CD40, and adhesion molecules: intercellular adhesion molecule (ICAM), vascular cell adhesion molecule (VCAM), and neural cell adhesion molecule (NCAM), which stabilize cell-cell interactions (**Figure 6F**). Detailed analysis of contact-dependent pairs (**Figure 6G**) showed that B cells in T1N+M0 possess an enhanced capacity for antigen presentation, evidenced by strong interactions between human leukocyte antigen (HLA)-class II molecules and CD4 on T cells, as well as HLA-class I engagement with NK cell receptors (e.g., KLRK1). Furthermore, as receivers of contact signals (**Figure 6H**), B cells in T1N+M0 benefited from a highly interactive network. Most notably, the CD40 ligand (CD40LG)-CD40 interaction was significantly upregulated, indicating productive T-B cell collaboration essential for B cell proliferation and class-switch recombination. This was complemented by adhesive stabilizers such as P-selectin glycoprotein ligand-1 (SELPLG)-L-selectin (SELL) and integrin beta-2 (ITGB2)-ICAM2.

Collectively, the exploratory analysis of secreted signaling pathway and contact-mediated signaling in **Figure 6** suggests that B cells in T1N+M0 BC orchestrate a superior anti-tumor immune response compared to those in T2N+M0. This advantage is driven by a multi-dimensional communication network: (a) a secretory profile rich in pro-inflammatory cytokines (TNF, MIF) and chemokines (CXCL13, CCL19) that recruits and activates immune effector cells; and (b) a contact-dependent landscape characterized by robust antigen presentation (MHC-TCR), effective co-stimulation (CD40-CD40L), and stable immune synapse formation. These exploratory findings highlight that the enhanced immune surveillance in T1N+M0 BC is structurally supported by a highly active and coordinated B cell communication axis, observations that require validation in larger, independent cohorts with adequate sample sizes.

## Discussion

By integrating data from the SEER database with comprehensive multi-omics profiling, this study suggests both the prognostic characteristics of OBC and the immunological mechanisms driving PT “occultation”. Our results demonstrate that the overall survival of OBC patients is comparable to T1 stage LN-positive BC, yet significantly superior to T2–3 stage counterparts. Convergent evidence from proteomic, transcriptomic, and scRNA-seq analyses identifies a robust B cell-mediated anti-tumor immune response as a defining biological hallmark of OBC. Specifically, within the T1 stage tumor microenvironment—a phenotype analogous to OBC—B cell infiltration is markedly enriched. These cells orchestrate extensive communication networks with T cells and NK cells via both secreted signaling and cell-cell contact pathways, establishing an immune milieu conducive to tumor clearance and conferring a significant survival advantage.

The tumor immune microenvironment is a critical determinant of clinical outcomes in BC, driving a paradigm shift toward immuno-oncology (21–23). Historically, the immunotherapeutic landscape has been dominated by a T-cell-centric view, with the vast majority of research focusing on CD8+ cytotoxic T lymphocytes as the primary executors of anti-tumor immunity (24–26). While this focus has yielded significant insights, the role of B cells has long been marginalized or underappreciated. However, emerging evidence has demonstrated that B cells are integral to the formation of tertiary lymphoid structures (27), production of tumor-specific antibodies (28), and, crucially, to antigen presentation and subsequent regulation of T cell responses (29). For instance, Tian et al. have identified B cell immune checkpoints like TIM-1 as potential immunotherapy targets (30). These multifaceted functions suggest that B cells possess a unique capacity to modulate the immune landscape. Recent studies by Sammut et al. (31) and Cole et al. (32) have further highlighted the role of B cell clonal persistence and cooperation with Th17 cells in maintaining durable tumor immunity. This re-evaluation underscores the necessity of understanding B cell-mediated mechanisms to fully exploit immunotherapeutic potential.

While our findings underscore the beneficial role of B cells in OBC, it is crucial to acknowledge the functional heterogeneity of B cell populations, as distinct subsets can exert divergent effects on tumor immunity. Regulatory B cells, for instance, are known to suppress anti-tumor responses by secreting immunosuppressive cytokines such as IL-10 and TGF-β, often correlating with poor clinical outcomes in breast cancer (33, 34). Conversely, other subsets like plasma cells and germinal center B cells are increasingly recognized as drivers of favorable prognosis, often serving as key components of tertiary lymphoid structures that sustain effector T cell activation (35, 36). In this study, scRNA-seq analysis revealed a robust anti-tumor response in T1N+ tumors, characterized by multi-faceted secretory signals (e.g., TNF, BAFF) and contact-dependent interactions (e.g., CD40, MHC). These findings suggest that B cell infiltrates in OBC are predominantly effector phenotypes rather than suppressive regulatory subsets, thereby underpinning the observed survival advantage.

Through the lens of Cancer Immunoediting theory (37), which delineates the phases of elimination, equilibrium, and escape, we propose a mechanistic explanation for the OBC paradox. We hypothesize that this process involves immune cells trafficking from the LN to the primary breast site. Concurrently, a highly invasive subclone characterized by high EMT disseminated to the LN prior to clearance (3). Despite immune surveillance within these niches, these evaded destruction via robust immune escape capabilities, enabling metastatic colonization. Consequently, OBC represents a unique spatial separation of immunoediting: the primary site undergoes immune-mediated completely elimination, while the metastatic site persists in a state of equilibrium or localized escape.

The dynamics of this immune regulation are further contextualized by the Cancer-Immunity Cycle framework proposed by Chen and Mellman, which describes the continuum of antigen release, presentation, T cell priming, trafficking, infiltration, and killing (38, 39). Our multi-omics data suggest that this cycle may operate with exceptional efficiency at the primary site of OBC. Exploratory proteomic analysis suggested a potential upregulation of B cell-related pathways and leukocyte transendothelial migration in OBC-LN, while transcriptomics confirmed enriched B cell infiltration in T1N+ tumors compared to T2–3N+ counterparts. scRNA-seq further revealed that within the T1N+M0 tumor microenvironment, B cells actively communicate with T cells via MHC engagement (MHC-I/MHC-II) and co-stimulatory signals (CD40-CD40LG). These interactions facilitate antigen presentation and T cell activation, propelling the Cancer-Immunity Cycle forward. Conversely, T2–3 tumors exhibit dominant stromal signaling (e.g., CXCL12-CXCR4) and suppressed B cell activity, indicating a disrupted cycle that facilitates immune escape. Notably, preliminary proteomic evidence also indicated enrichment of MDSCs in OBC-LN; these cells suppress lymphocyte-mediated cytotoxicity, potentially explaining the persistence of metastasis in this niche despite systemic immune surveillance (40–42).

Furthermore, we propose a hypothesis that the superior prognosis of OBC is driven not only by the clearance of the PT but also by sustained humoral immunity that controls distant dissemination. The conventional linear model of metastasis (primary-LN-distant) has been challenged by phylogenetic evidence from Venet et al. (43), suggesting that distant dissemination is frequently driven by direct seeding from PT, rather than a sequential event following LN metastasis. In this context, the activated B cell response in OBC may provide systemic immunosurveillance that suppresses these direct metastatic events. This aligns with our observation that B cells in T1N+M0 tumors utilize secreted signals (e.g., CXCL13, BAFF) and cell-cell contact to maintain an active immune niche (44–46), thereby restricting the outgrowth of occult micrometastases.

This study is subject to several limitations. First, the rarity of OBC resulted in limited sample sizes for proteomic analysis. Specifically, protein profiles were derived from a comparison involving only two patients with OBC and two matched patients with non-OBC. Consequently, while these findings offer valuable exploratory insights into the molecular landscape of OBC, the small sample size inherently limits the statistical power and generalizability of these results, necessitating cautious interpretation and validation in larger independent cohorts. Furthermore, the reliance on scRNA-seq data exclusively from TNBC patients restricts the generalizability of our findings to other molecular subtypes. Second, the absence of a detectable primary tumor necessitated the use of T1N+M0 patients as a surrogate model, introducing an inherent degree of assumption. Third, the study is primarily based on correlative multi-omics data and lacks functional *in vitro* and *in vivo* experiments to directly validate the causal role of B cells in primary tumor clearance. Finally, despite PSM, the retrospective nature of the SEER database analysis precludes the complete exclusion of selection bias and unmeasured confounding factors.

## Conclusion

Our findings suggest that the favorable prognosis of OBC may be linked to a B cell-centric immune network, which potentially contributes to the clearance of the PT and aids in maintaining systemic control over disseminated disease. These exploratory results underscore the potential of B cells as prognostic biomarkers and therapeutic targets in BC, pending functional validation in larger cohorts.

## Data Availability

The datasets presented in this study can be found in online repositories. The names of the repository/repositories and accession number(s) can be found in the article/**Supplementary Material**.

## Supplementary material

Supplementary Figure 1 The inclusion and exclusion criteria for patients who were enrolled from the SEER dataset.

Supplementary Figure 2 Venn diagram and functional enrichment of downregulated DEPs in proteomic profiling (OBC-LN: n=2; non-OBC-LN: n=2; and non-OBC-PT n=2).

Supplementary Figure 3 KEGG pathway enrichment of OBC-LN-specific upregulated proteins and immune infiltration analysis.

Supplementary Figure 4 Quality control and cell clustering of scRNA-seq data.

Supplementary Figure 5 Heatmaps of differential cell-cell communication involving B cells in T1N+M0 versus T2N+M0 BC.

Supplementary Table 1 Clinicopathological characteristics of OBC patients and matched non-OBC patients selected for proteomics analysis.

Supplementary Table 2 Proteomic profiles of OBC and non-OBC.

Supplementary Table 3 Clinicopathological characteristics of breast cancer patients for transcriptome analysis.

Supplementary Table 4 GO enrichment analysis of OBC-LN-specific up-regulated DEPs.

Supplementary Table 5 GO enrichment analysis of non-OBC-LN-specific up-regulated DEPs.

Supplementary Table 6 GO enrichment analysis of up-regulated DEPs shared by OBC-LN and non-OBC-LN.

Supplementary Table 7 KEGG enrichment analysis of OBC-LN-specific up-regulated DEPs.

## Glossary

AJCC: American Joint Committee on Cancer
ALN: Axillary lymph node
APRIL: A proliferation-inducing ligand
BAFF: B-cell activating factor
BC: Breast cancer
BH: Benjamini-Hochberg
BP: Biological Process
CC: Cellular Component
CD40LG: CD40 ligand
CIs: Confidence intervals
COD: Cause of death
DEPs: Differentially expressed proteins
EMT: Epithelial-mesenchymal transition
EOD: Extent of Disease
FDR: False discovery rate
FFPE: Formalin-fixed and paraffin-embedded
GEO: Gene Expression Omnibus
GO: Gene Ontology
HLA: Human leukocyte antigen
HR: Hormone receptor
HRs: Hazard ratios
ICAM: Intercellular adhesion molecule
IHC: Immunohistochemical
ITGB2: Integrin beta-2
KEGG: Kyoto Encyclopedia of Genes and Genomes
LGALS9: Galectin-9
LN: Lymph node
METABRIC: Molecular Taxonomy of Breast Cancer International Consortium
MF: Molecular Function
MHC: Major histocompatibility complex
MIF: Migration inhibitory factor
moDCs: monocyte-derived dendritic cells
NCAM: Neural cell adhesion molecule
NK cells: Natural killer cells
Non-OBC: Non-occult breast cancer
Non-OBC-LN: Paired metastatic lymph node from non-occult breast cancer
Non-OBC-PT: Primary tumors from non-occult breast cancer
OBC: Occult breast cancer
OBC-LN: OBC metastatic LN
OS: Overall survival
PB: Peripheral blood
PCA: Principal component analysis
PSM: Propensity score matching
PT: Primary tumor
scRNA-seq: Single-cell RNA sequencing
SEER: Surveillance Epidemiology and End Results
SELL: L-selectin/
SELPLG: P-selectin glycoprotein ligand-1
SMD: Standardized mean difference
SNN: Shared nearest neighbor
ssGSEA: Single-sample gene set enrichment analysis
STROBE: Strengthening the Reporting of Observational Studies in Epidemiology
TNBC: Triple-negative breast cancer
TNF: Tumor necrosis factor
TPM: Transcripts per million
UMAP: Uniform Manifold Approximation and Projection
UMIs: Unique molecular identifiers
VCAM: Vascular cell adhesion molecule.
