# Supplementary material for: B cell-mediated immune surveillance defines the favorable prognosis of occult breast cancer: a multi-omics study
Source: Front Immunol. 2026 Apr 17;17:1813674. doi: 10.3389/fimmu.2026.1813674 (PMC13133082; doi:10.3389/fimmu.2026.1813674)
Supplement: Supplementary Figure 1 — The inclusion and exclusion criteria for patients who were enrolled from the SEER dataset. [file DataSheet1.pdf]

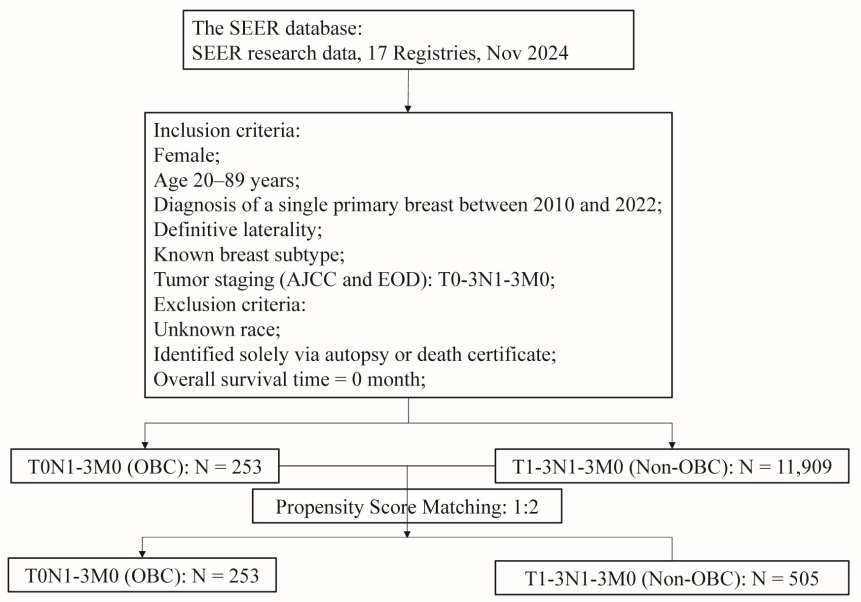

Figure S1. The inclusion and exclusion criteria for patients who were enrolled from the SEER dataset. The flowchart was constructed based on the inclusion and exclusion criteria detailed in the Materials and methods section.

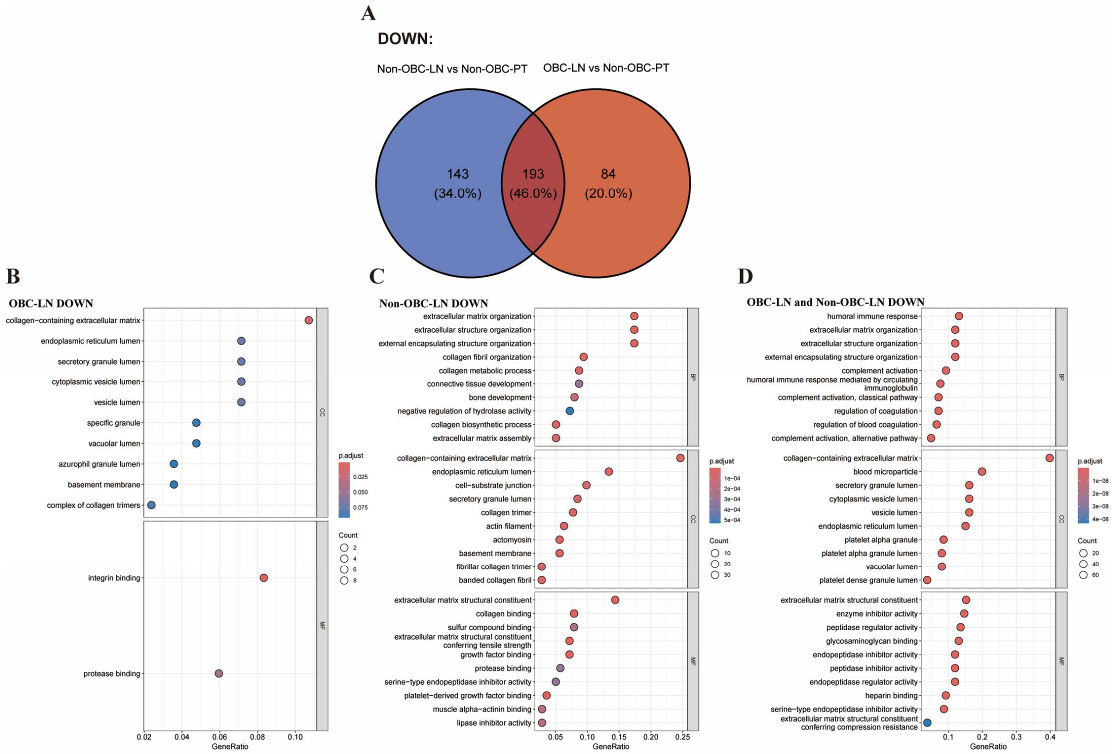

Figure S2. Venn diagram and functional enrichment of downregulated DEPs in proteomic profiling (OBC-LN: n=2; non-OBC-LN: n=2; and non-OBC-PT n=2). **(A)** Venn diagrams illustrating the unique and shared down-regulated DEPs between OBC-LN and non-OBC-LN, generated using the R ggvenn package. **(B-D)** Bubble plots displaying GO enrichment analysis of down-regulated DEPs subsets. DEPs were identified using the R limma package with thresholds of $|log_2FC| > 0.5$ and $P < 0.05$. GO enrichment was performed using the R clusterProfiler package, with statistical significance defined as a Benjamini-Hochberg adjusted P-value < 0.05. **(B)** OBC-LN-specific down-regulated DEPs. **(C)** non-OBC-LN-specific down-regulated DEPs. **(D)** Down-regulated DEPs shared by both OBC-LN and non-OBC-LN.

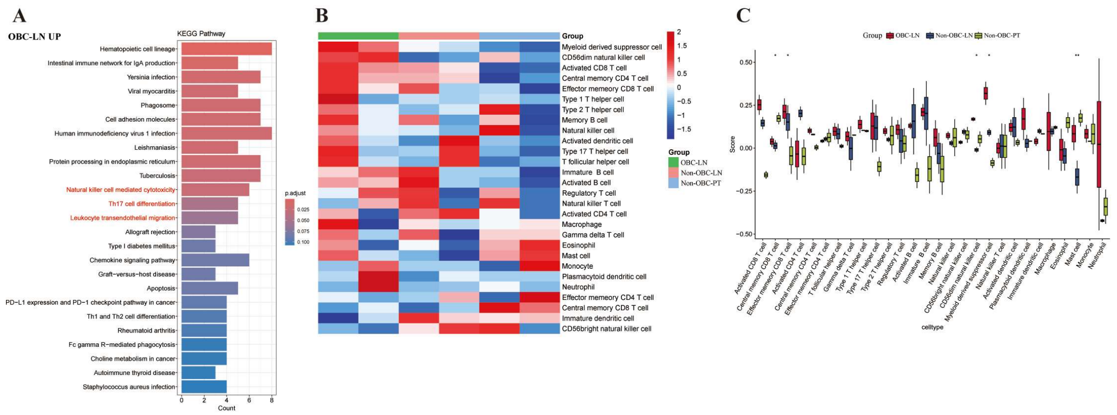

Figure S3. KEGG pathway enrichment of OBC-LN-specific upregulated proteins and immune infiltration analysis. **(A)** KEGG pathway enrichment analysis of OBC-LN-specific upregulated DEPs, performed using the R clusterProfiler package based on KEGG Release 116.0. **(B, C)** Immune cell infiltration quantified by single-sample gene set enrichment analysis (ssGSEA) using the R GSVA package. Statistical differences among OBC-LN (n=2), non-OBC-LN (n=2), and non-OBC-PT (n=2) groups were assessed using the Kruskal-Wallis test ($P < 0.05$). * indicates: $P < 0.05$; ** indicates: $P < 0.01$.

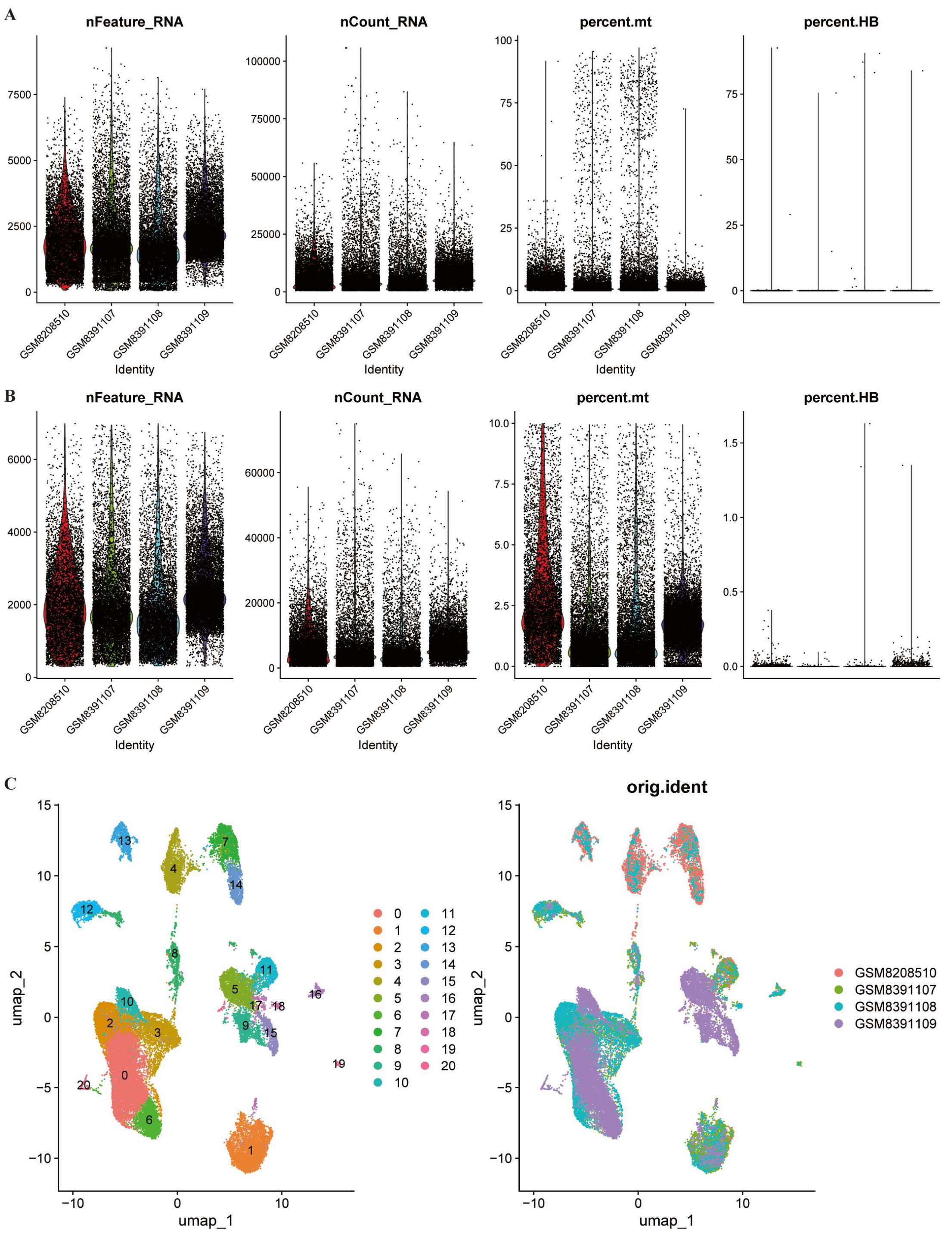

Figure S4. Quality control and cell clustering of scRNA-seq data. **(A, B)** Scatter plots showing quality control metrics for each sample (GSM8208510, GSM8391107–1109). Low-quality cells were filtered using the R Seurat package based on the following criteria: detected gene counts outside 300–7,000, mitochondrial gene percentage > 10%, and hemoglobin gene percentage > 3%. **(C)** UMAP visualization of cell clusters (left) and sample origin (right). Data were normalized using the LogNormalize method, batch effects were corrected using the Harmony algorithm, and cells were clustered using a shared nearest neighbor (SNN) algorithm with a resolution of 0.5.

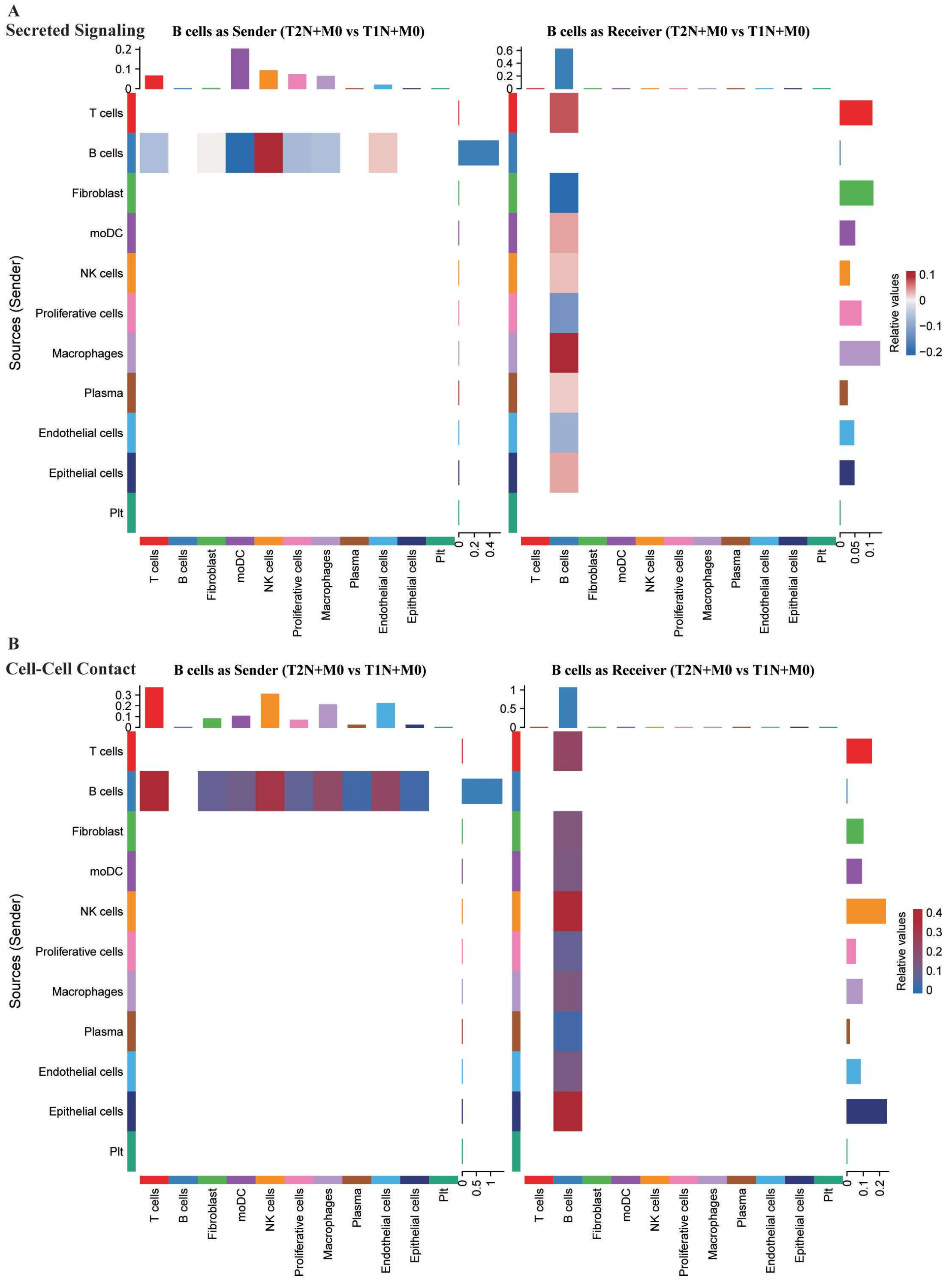

Figure S5. Heatmaps of differential cell-cell communication involving B cells in T1N+M0 versus T2N+M0 BC. Heatmaps showing the relative communication strength of secreted signaling (A) and cell-cell contact (B) pathways, with B cells as senders (left) and receivers (right). Comparative analysis was performed using the R CellChat package by merging objects from T1N+M0 and T2N+M0 samples. Visualization was generated using the ComplexHeatmap framework.
